# Regulation of von-Willebrand Factor Secretion from Endothelial Cells by the Annexin A2-S100A10 Complex

**DOI:** 10.3390/ijms19061752

**Published:** 2018-06-13

**Authors:** Anna Holthenrich, Volker Gerke

**Affiliations:** Institute of Medical Biochemistry, Centre for Molecular Biology of Inflammation, University of Münster, Von-Esmarch-Strasse 56, 48149 Münster, Germany; a_holt12@uni-muenster.de

**Keywords:** calcium signaling, endothelial cells, exocytosis, hemostasis, membrane trafficking

## Abstract

Endothelial cells serve as gatekeepers of vascular hemostasis and local inflammatory reactions. They can rapidly respond to changes in the environment, caused, for example, by blood vessel injury, tissue damage or infection, by secreting in a strictly regulated manner factors regulating these processes. These factors include adhesion receptors for circulating leukocytes and platelets, P-selectin and von-Willebrand factor (VWF) that are stored in specialized secretory granules of endothelial cells, the Weibel-Palade bodies (WPB). Acute exposure of these adhesion molecules converts the endothelial cell surface from an anti-adhesive state enabling unrestricted flow of circulating blood cells to an adhesive one capable of capturing leukocytes (through P-selectin) and platelets (through VWF). While these are important (patho)physiological responses, compromised or dysregulated WPB secretion can cause pathologies such as excessive bleeding or vascular occlusion. Several factors are involved in regulating the exocytosis of WPB and thus represent potential targets for therapeutic interventions in these pathologies. Among them, the annexin A2 (AnxA2)-S100A10 complex has been shown to participate in the tethering/docking of secretion-competent WPB at the plasma membrane, and interference with AnxA2/S100A10 expression or complex formation significantly reduces acute WPB exocytosis and VWF release. Thus, developing specific means to efficiently block AnxA2-S100A10 complex formation in endothelial cells could lead to novel avenues towards interfering with acute vascular thrombosis.

## 1. Endothelial Cells as Regulators of Vascular Homeastasis

Endothelial cells represent the inner lining of blood vessels and thus form the principal border between vasculature and tissues. As such, they regulate the transport of metabolites, other small molecules and also cells that can penetrate the endothelial barrier, either trans- or paracellularly, involving complex transport systems and a dynamic regulation of the endothelial cell-to-cell junctions. In addition to serving functions as a physical barrier, endothelial cells also control vascular homeostasis by supplying the blood with factors that regulate thrombosis, fibrinolysis and local inflammatory events. The release of these factors is tightly regulated at the level of the endothelial cells. This is particularly evident when the adhesive properties of the endothelial surface are considered. While resting endothelial cells present an anti-coagulant surface to circulating cells of the vasculature, endothelial activation converts this surface to a pro-coagulant and pro-inflammatory one capable of capturing platelets and leukocytes. This transition critically depends on the acute and tightly regulated exposure of adhesion molecules, most notably von-Willebrand factor (VWF) and P-selectin, which serve as receptors for platelets and leukocytes, respectively, and are stored in large secretory granules of endothelial cells, the Weibel-Palade bodies (WPB) discovered in electron microscopy sections of arterial endothelia by Ewald Weibel and George Palade more than 50 years ago [1].

VWF is a multimeric glycoprotein. It binds several collagens, including types I, II, III, IV, V and VI that become exposed in the subendothelial matrix once the endothelial blood vessel lining is damaged. VWF also interacts with platelet glycoprotein Ib and other receptors on activated platelets. To efficiently capture platelets at sites of vessel injury, VWF has a unique property; it forms elongated strings of covalently linked concatamers that can consist of more than 100 VWF molecules and can span a length of more than 100 μm (for review, see [2,3,4,5]). Inside endothelial cells, VWF undergoes a complex maturation process. Following synthesis and dimerization in the endoplasmic reticulum, it is subject to cleavage and multimerization reactions in the Golgi apparatus and the trans Golgi network (TGN). At this stage, defined numbers of VWF molecules are packaged into ministack Golgi cisternae from where early, immature WPB emerge [6]. The WPB then acquire additional components from the endosomal system as well as cytoplasmic proteins, most notably the small GTPase Rab27a, thus sharing some characteristics with lysosome-related organelles such as pigment-storing melanosomes. Within WPB, VWF undergoes further maturation, including continued multimerization (for review, see [4,7]). The tightly packed and highly ordered VWF multimers dictate the unique form of WPB, which are elongated, rod-like organelles [4]. Following exocytosis, VWF unfurls at WPB fusion sites and forms long strings promoted by shear forces of the circulation [8]. VWF maturation and secretion, and thus the levels of released VWF are compromised in several human disorders. They include von-Willebrand disease, the most common inherited bleeding disorder in which patients lack functional, high molecular weight forms of VWF [9], and thrombotic thrombocytopenic purpura, which is characterized by vascular occlusions resulting from highly elevated VWF levels in the vasculature [10].

P-selectin is the other major adhesion receptor stored in WPB. Following inflammatory stimulation of endothelial cells, P-selectin is externalized via regulated WPB exocytosis and then provides binding sites for P-selectin ligands on the surface of circulating leukocytes. This initiates their tethering and rolling as a prerequisite for firm adhesion and subsequent extravasation. The tetraspanin CD63 functions as a cofactor of P-selectin, presumably through stabilization of larger P-selectin clusters at the cell surface [11]. In endothelial cells, CD63 is found to be associated with late endosomes (for review, see [12]) and is also present in WPB from where it is released to the cell surface upon endothelial stimulation. Little is known about the transport of endosomal CD63 to WPB, but one annexin protein, annexin A8 (AnxA8), has been shown to be crucially involved in this step [13,14].

As the controlled release by endothelial cells of VWF and P-selectin/CD63 is crucial for the proper reaction to vessel injury and local inflammatory stimuli, understanding the mechanism of acute WPB exocytosis is highly relevant also for translational medical approaches. Exocytosis of WPB can be triggered by two main groups of agonists, those acting by elevating the free concentration of cytosolic Ca^2+^ such as histamine and thrombin, and those acting by raising cyclic adenosine monophosphate (cAMP) levels such as epinephrin and vasopressin. Significant phenomenological differences exist between the Ca^2+^ and cAMP dependent release pathways but fairly little is known about underlying mechanisms and cross-talk between the pathways.

## 2. Mechanism of Regulated WPB Exocytosis

Following biogenesis and microtubule-dependent transport to the cell periphery, WPB are anchored in the cortical actin cytoskeleton via a tripartite complex comprising WPB-associated Rab27a, the Rab27a effector MyRIP and myosin Va [15,16,17]. VWF in the cortically anchored WPB undergoes final maturation steps that are required to generate the long VWF multimers serving as strings for efficient platelet recruitment after WPB exocytosis [15,16]. Interference with cortical anchorage of WPB, e.g., by depletion of MyRIP or myosin Va, leads to a redistribution of WPB to the perinuclear region. Interestingly, a similar redistribution of some peripheral WPB is seen following stimulation of endothelial cells, in particular with cAMP-elevating agonists [18], suggesting that WPB are released from the cortical anchorage in preparation of the final plasma membrane fusion. However, it is not known how this release is triggered, although it could be speculated that the GTPase Rab27a acts as an important switch.

Similar to other fusion events, the final fusion of mature WPB with the plasma membrane requires the activity of *N*-ethylmaleimide-sensitive factor (NSF) and proteins of the soluble NSF attachment protein receptor (SNARE) family [19]. One SNARE complex involved in Ca^2+^-dependent WPB exocytosis consists of plasma membrane-resident syntaxin-4 and SNAP-23 and the WPB-associated VAMP3 that possibly function in conjunction with accessory proteins such as syntaxin binding protein 1 (StxBP1) and StxBP5 (also known as Munc18-1 and -5) [20,21,22]. Other factors functioning in the Ca^2+^ and/or cAMP dependent exocytosis of WPB have also been reported (for review, see [23]). They include the small GTPases RalA and Rap1 and their regulators RalGDS and Epac [24] as well as several Rab family proteins, in particular Rab3 isoforms and Rab27a, which are acquired by WPB during their maturation [25,26,27] and Rab15 and Rab32, which are likely to function at a late stage of WPB exocytosis [28,29].

Rab27a is a particularly relevant Rab GTPase in WPB formation and secretion. It acts by serving together with MyRIP and myosin Va in tethering WPB at the cortical cytoskeleton, thereby inhibiting premature WPB exocytosis. Rab27a can also interact with another effector, synaptotagmin-like protein 4a (Slp4a), resulting in a promotion of Ca^2+^ dependent WPB exocytosis, and it has been proposed that the fractional occupancy of WPB-associated Rab27a by Slp4a and MyRIP controls the probability of WPB cargo release with the former favoring exocytosis and the latter cortical anchorage [27]. Another effector of Rab27a also expressed in endothelial cells is Munc13-4, a member of the Munc13 family of proteins thought to function as tethering and/or priming factors in SNARE-mediated exocytotic fusion events (for review, see [30]). Munc13-4 is localized to WPB and the plasma membrane of human endothelial cells and acts as a positive regulator of evoked WPB exocytosis [28,31]. Interestingly though, albeit being a known Rab27a effector, the recruitment of Munc13-4 to WPB does not require Rab27a and Munc13-4 acts in WPB exocytosis in a Rab27a independent manner [31]. High resolution live cell imaging revealed that Munc13-4 is most likely a long sought-after tethering factor in Ca^2+^ dependent WPB exocytosis. Following secretagogue stimulation, Munc13-4 clusters at sites of WPB-plasma membrane contact prior to the actual membrane fusion event and this clustering is mediated by the two Ca^2+^ sensitive lipid- and protein-binding C2 domains of the protein [31].

The C2 domain interactions of Munc13-4 are most likely of crucial relevance in the pre-fusion tethering of WPB and the establishment of membrane platforms that can serve as designated fusion sites. While the N-terminal C2A domain can interact with different syntaxins including those involved in WPB-plasma membrane fusion, the C-terminal C2B domain can bind Ca^2+^ dependently to acidic phospholipids possibly enriched at sites of WPB exocytosis and thereby assist docking of WPB to certain plasma membrane domains [32]. It is tempting to speculate that such lipid binding is connected to the phospholipase D1-mediated generation of enhanced levels of phosphatidic acid (PA) in the plasma membrane of secretagogue-stimulated endothelial cells [33]. Another protein recruited to acidic phospholipids enriched in the membrane of secretagogue-stimulated endothelial cells is the annexin family member AnxA2 [34]. AnxA2 is known to reside in a tight heterotetrameric complex with S100A10 and in this complex most likely assists membrane tethering of WPB-bound Munc13-4 as S100A10 was identified as a direct interactor of the C2 domains of Munc13-4 [31]. Intriguingly, siRNA-mediated downregulation of AnxA2 and S100A10 significantly interferes with the histamine-evoked and Ca^2+^ dependent recruitment of Munc13-4 to WPB fusion sites [31]. This suggests that the AnxA2-S100A10 complex plays an important role in acute WPB exocytosis by establishing plasma membrane platforms rich in acidic phospholipids and serving as a docking factor for WPB-enriched Munc13-4, thereby establishing a link between fusion-competent WPB and the plasma membrane. Thus, specifically interfering with this activity of AnxA2-S100A10 could provide a means of reducing the acute release of pro-thrombotic WPB contents also in pathological scenarios.

## 3. The AnxA2-S100A10 Complex: Structural Insights and Regulation of Complex Formation

AnxA2 is one of the annexin family members known to specifically interact with EF hand-type proteins of the S100 family (for review, see [35,36,37,38]). S100 proteins are characterized structurally by two consecutive Ca^2+^ binding EF hands that are connected by a short linker region and flanked by relatively short N- and C-terminal extensions. They act as molecular switches in the transmission of Ca^2+^ signals; in response to elevated cytosolic Ca^2+^ levels, S100 proteins show a conformational change that leads to the exposure of hydrophobic surfaces acting as binding sites for downstream effectors, which are regulated in their localization and/or activity by the S100 binding (for review, see [39,40]). Although interactions of AnxA2 with Ca^2+^ bound S100A4 and S100A11 have been reported [41,42,43,44], the best described binding partner of AnxA2 among the S100 proteins is S100A10 (for review, see [45,46]). S100A10, also known as p11, is unique within the S100 family, as it shows amino acid changes and deletions in both EF hands that render the protein incapable of binding Ca^2+^. These changes establish a conformation equivalent to other Ca^2+^ bound S100 proteins, i.e., with hydrophobic binding sites exposed, and S100A10 can therefore be considered a permanently active S100 protein capable of interacting with its effector proteins, in particular AnxA2, irrespective of the presence or absence of Ca^2+^ [47].

The interaction between AnxA2 and S100A10 has been well described at the molecular level. In the annexin chain, the S100A10 binding site is confined to the N-terminal 10 to 12 residues that form an amphiphatic α-helix with the hydrophobic side representing the major binding surface for S100A10. Importantly, acetylation of the N-terminal serine residue is required to stabilize the helix and thus required for high affinity S100A10 binding [47,48,49]. In the S100A10 chain, binding to AnxA2 requires residues in the unique C-terminal extension following the second EF hand [47,50]. As S100A10 forms obligatory dimers, two AnxA2 binding sites exist per dimeric S100A10 and the AnxA2-S100A10 complex is tetrameric in nature. Most cells analyzed in this respect so far seem to express both, monomeric as well as heterotetrameric, S100A10-complexed AnxA2, as, for example, analyzed by gel filtration of cell lysates [51]. The binding between AnxA2 and S100A10 is of high affinity, with a dissociation constant in the nanomolar range [48] and, as pointed out above, complex formation occurs Ca^2+^ independently. Therefore, the presence of an appreciable pool of monomeric AnxA2 within cells has to be the consequence of either limited S100A10 expression and/or posttranslational modifications that weaken the interaction. The latter could involve proteolytic shortening of the S100A10 chain as already removal of the C-terminal five residues almost completely abrogates AnxA2 binding [50]. However, so far, such selective proteolysis has not been described in vivo, although C-terminal ubiquitination and subsequent proteolysis has been shown to result in the complete proteasome-mediated degradation of non-complexed S100A10 [52].

Limited proteolysis of AnxA2 could also affect complex formation with S100A10. As the S100A10 binding site resides in the first 10 to 12 residues of the AnxA2 chain, N-terminal proteolysis of only a few amino acid residues results in a truncated AnxA2 form incapable of binding S100A10 [48]. However, although in vitro studies revealed that the N-terminal domain of AnxA2 is sensitive to limited proteolysis, the existence of such selective proteolytic processing within cells has not been demonstrated so far. In contrast, two other posttranslational modifications that could affect S100A10 binding have been shown to occur in the first 12 residues of AnxA2, specifically at Ser-1 and Ser-11. The N-terminal serine residue is acetylated and this acetylation is required for high affinity S100A10 binding [53]. However, so far, all AnxA2 species purified from mammalian cells or tissues that were assessed with respect to this modification showed N-terminal acetylation (see for example, [54]), suggesting that acetylation is an obligatory modification and not subject to regulation. Another modification in the N-terminal sequence of AnxA2 is a phosphorylation at Ser-11, which can be catalyzed by protein kinase C (PKC) (for review, see [55]). Interestingly, phosphorylation at this site interferes with high affinity S100A10 binding as revealed by direct interaction studies involving purified proteins [56,57]. Moreover, PKC-dependent phosphorylation as well as protein kinase A (PKA)-triggered dephosphorylation of AnxA2 by a calcineurin-like phosphatase have been demonstrated to regulate complex formation with S100A10 in airway epithelial and endothelial cells [58,59]. Collectively, these data suggest that phosphorylation at Ser-11 is a means of regulating AnxA2-S100A10 complex formation in cells, with high affinity interaction requiring a non-phosphorylated Ser-11.

## 4. The AnxA2-S100A10 Complex in Regulated Secretion

While many annexins have been implicated in the regulation of membrane trafficking steps along the endocytic and exocytotic pathways, AnxA2 in complex with S100A10 has been clearly shown to participate in a specific set of (typically Ca^2+^) regulated secretory events. These include the exocytotic release of chromaffin granules from neuroendocrine chromaffin cells, the exocytosis of lamellar bodies from alveolar epithelial cells and WPB exocytosis in endothelial cells.

AnxA2 was in fact first described by Carl Creutz as a soluble protein that can bind to chromaffin granule membranes in the presence of Ca^2+^, therefore it was initially also termed chromobindin [60]. Later work, mainly by the groups of Marie-France Bader and Sylvette Chasserot-Golaz, showed that it is required for the Ca^2+^ evoked exocytosis of these granules and that this activity requires complex formation with S100A10 [61]. In adrenergic chromaffin cells, AnxA2 is recruited to the subplasmalemmal region following nicotinic stimulation where it forms the AnxA2-S100A10 heterotetrameric complex in a manner that also involves PKC phosphorylation at Ser-25 [62]. In the cortex of stimulated chromaffin cells, AnxA2-S100A10 most likely participates in the formation/stabilization of membrane domains and actin bundling at sites of granule fusion [63,64,65]. In type II alveolar epithelial cells, work by the Liu group has shown that AnxA2, but not other annexins including AnxA1, A5 and A6, stimulates the exocytosis of lamellar bodies from permeabilized type II cells and that this activity of AnxA2-S100A10 is enhanced by membrane-bound arachidonic acid [66,67].

In endothelial cells, AnxA2-S100A10 is involved in only a subset of the secretory events elicited by Ca^2+^ elevation. Using a whole cell patch-clamp approach in bovine aortic endothelial cells, it was initially shown that the majority but not all of the Ca^2+^ dependent exocytotic events, as measured by an increase in membrane capacitance, can be inhibited by dissociation of the AnxA2-S100A10 complex. This dissociation was achieved by the intracellular delivery through the patch pipette of synthetic peptides that corresponded to the N-terminal 14 residues of AnxA2 and thus competed with endogenous AnxA2 for S100A10 binding. In human umbilical vein endothelial cells (HUVEC), it was then shown that the Ca^2+^ dependent exocytotic events affected by this complex disruption and also by siRNA-mediated knockdown of AnxA2 or S100A10 were those of WPB releasing VWF but not those mediating the Ca^2+^ dependent secretion of tissue-type plasminogen activator [26]. Similar to the scenario in chromaffin cells, it appears that the AnxA2-S100A10 complex is involved in generating and/or stabilizing membrane platforms rich in acidic phospholipids such as PS and PA that represent docking/fusion sites of WPB (see also above). Such activity of the AnxA2-S100A10 complex is supported by in vitro studies using purified proteins and giant unilamellar vesicles (GUVs) or solid-supported membranes analyzed by atomic force microscopy that revealed an AnxA2-S100A10 mediated clustering of negatively charged phospholipids into larger domains [68,69,70]. AnxA2-S100A10 concentrated at WPB docking sites most likely also functions in recruiting other factors, in particular the WPB associated Munc13-4 through the direct Munc13-4/S100A10 interaction, and thereby constitutes part of a physical link in the course of WPB tethering [31]. Interestingly, this action of AnxA2-S100A10 in acute WPB exocytosis appears to be regulated by the control of complex formation through AnxA2 phosphorylation. As mentioned above, phosphorylation at Ser-11, a site very close to the actual S100A10 binding sequence, can compromise complex formation. In endothelial cells, this phosphorylation can be triggered by plasmin-induced activation of PKC [59] and has been shown to result in a dissociation of the AnxA2-S100A10 heterotetramer. Conversely, dephosphorylation at this site stabilizes the AnxA2-S100A10 complex and this has been shown to occur in endothelial cells following stimulation with agonists raising the cellular cAMP levels that also elicit WPB exocytosis. Elevation of intraendothelial cAMP triggers the PKA dependent activation of a calcineurin-like phosphatase that dephosphorylates AnxA2 at Ser-11 and thereby promotes complex formation with S100A10. Moreover, depletion and mutant rescue experiments revealed that only AnxA2 mutants capable of complex formation with S100A10 supported the cAMP-dependent exocytosis of WPB and thus the evoked secretion of VWF [34]. Therefore, it appears that, in endothelial cells, AnxA2-S100A10 complex formation and membrane translocation are required for the formation of Munc13-4-enriched WPB tethering sites in the course of WPB exocytosis.

## 5. Means of Disrupting the AnxA2-S100A10 Complex

Given the important role of AnxA2-S100A10 in mediating the exocytosis of mature WPB and thus the release of ultralong VWF fibers, it is reasonable to assume that interfering with AnxA2-S100A10 complex formation can represent a highly specific tool of controlling VWF secretion and thus the thrombogenic potential of activated endothelial cells. Different means of experimentally altering complex formation have been described. As the S100A10 binding site in AnxA2 is confined to the N-terminal 10 to 12 residues, peptides comprising this sequence have been used successfully to disrupt the complex (see above). The amphipathic helix formed by this sequence has a hydrophobic side that represents the principal interaction surface and binds to a hydrophobic pocket formed by the S100A10 dimer [47]. Hydrophobic residues in AnxA2 providing the prime contact have been identified. They include Val-3, Ile-6, Leu-7 and Leu-10 as well as the N-terminal acetyl group [53]. Therefore, mutant peptides containing single or double replacements of these hydrophobic residues or lacking the N-terminal acetyl groups can be used as highly specific controls in peptide-mediated complex disruption experiments. These mutant peptides show an affinity for S100A10 that is at least two orders of magnitude lower than that of the wild-type acetylated peptide and that do not dissociate preformed AnxA2-S100A10 complexes in cellular environments. Experiments using these mutant peptides as controls have indeed revealed the highly specific role of the complex in endothelial WPB exocytosis [26,71] and in the regulation of plasma membrane-resident ion channels in epithelial cells [58,72]. However, while these N-terminal AnxA2 peptides show a high affinity interaction and are highly specific for S100A10 binding and thus in complex disruption, their applicability in live cell systems or even organisms is rather limited as they cannot penetrate the cell membrane. Their use in cell culture systems required microinjection or transient membrane opening, e.g., by whole cell patching. Therefore, other means of interfering in a specific manner with AnxA2-S100A10 complex formation, ideally by using membrane-permeable small chemical inhibitors, are desirable.

Some chemical inhibitors of the AnxA2-S100A10 complex have been described by the group of Lodewijk Dekker (for review, see [46]). Following a structure-based random high-throughput docking approach, substituted 3-hydroxy-1*H*-pyrrol(5*H*)-2-one analogs were identified as inhibitors of AnxA2 binding to S100A10 [73]. An alternative, ligand-guided screen identified substituted 1,2,4-triazole analogs as competitive inhibitors of the AnxA2-S100A10 complex formation [74]. Docking studies revealed that both kinds of chemical inhibitors can interact with binding pockets normally occupied by the acetyl, valine and leucine moieties of the N-terminal AnxA2 sequence [73,74]. Based on the triazoles further analogs were synthesized to improve specificity and binding affinity and shown to disrupt native AnxA2-S100A10 complexes [75]. Using the 3-hydroxy-1*H*-pyrrol(5*H*)-2-ones and the 1,2,4-triazole analogs as lead compounds, further developments towards the generation of even more specific and potent small molecule inhibitors of the AnxA2-S100A10 complex can be expected. These compounds would be ideal tools to specifically interfere with activities mediated by the AnxA2-S100A10 complex, for instance the docking/fusion of endothelial WPB in the course of prothrombotic responses.

## 6. Conclusions

Endothelial cells are important regulators of thrombotic and fibrinolytic events in the vasculature. Upon activation, they release VWF and P-selectin that confer adhesiveness to the endothelial cell surface and act in promoting local platelet and leukocyte recruitment. The secretion of these surface receptors is tightly regulated and mediated by the controlled exocytosis of WPB that represent the storage organelles for VWF and P-selectin. Once deregulated, the excessive or diminished secretion of VWF and P-selectin can result in pathological scenarios, for example excessive bleeding, compromised immunological responses or vascular occlusion. Therefore, understanding the mechanism of WPB exocytosis and establishing modes to specifically inhibit or promote WPB exocytosis and thus P-selectin and VWF release is highly relevant also for translational medical approaches. As the AnxA2-S100A10 complex serves a specific and important function in the course of WPB exocytosis, interfering with complex formation can be considered a highly selective means of controlling VWF and P-selectin levels in the vasculature, possibly also in pathological scenarios.
